# Label Propagation with *α*-Degree Neighborhood Impact for Network Community Detection

**DOI:** 10.1155/2014/130689

**Published:** 2014-11-26

**Authors:** Heli Sun, Jianbin Huang, Xiang Zhong, Ke Liu, Jianhua Zou, Qinbao Song

**Affiliations:** ^1^School of Electronic and Information Engineering, Xi'an Jiaotong University, Xi'an 710049, China; ^2^State Key Laboratory for Novel Software Technology, Nanjing University, Nanjing 210023, China; ^3^School of Software, Xidian University, Xi'an 710071, China; ^4^School of Computer Science and Technology, Xidian University, Xi'an 710071, China

## Abstract

Community detection is an important task for mining the structure and function of complex networks. In this paper, a novel label propagation approach with *α*-degree neighborhood impact is proposed for efficiently and effectively detecting communities in networks. Firstly, we calculate the neighborhood impact of each node in a network within the scope of its *α*-degree neighborhood network by using an iterative approach. To mitigate the problems of visiting order correlation and convergence difficulty when updating the node labels asynchronously, our method updates the labels in an ascending order on the *α*-degree neighborhood impact of all the nodes. The *α*-degree neighborhood impact is also taken as the updating weight value, where the parameter impact scope *α* can be set to a positive integer. Experimental results from several real-world and synthetic networks show that our method can reveal the community structure in networks rapidly and accurately. The performance of our method is better than other label propagation based methods.

## 1. Introduction

With the emergence of all kinds of networks such as the Internet, social networks, and organic molecules networks human beings have stepped into the network era. Detection of community structure in real networks has important theoretical significance and high application value. For example, the community structure of social networks [1] can reveal groups of the same interests, opinions, or beliefs and the communities in a bimolecular network can represent the different functional modules [2–5].

At present, many kinds of algorithms for community detection in complex networks have been proposed, such as hierarchical clustering, modularity optimization, and spectral clustering [6–12]. However, some of the existing methods suffer from the problems of prior information requirements, parameter sensitivity, poor time efficiency, and so forth. In 2007, a label propagation algorithm was proposed by Raghavan et al. [13], called LPA, which can detect the intrinsic communities in a network without prior information. Because of its simplicity, high speed, and time efficiency, LPA has drawn much attention recently. LPA and most improved algorithms of it update the label of each node in an asynchronous way until a general consensus is reached. Each node updates its label based on its adjacent neighbor label status, and different nodes have the same influence on its neighborhood [13–16]. As a result, the labels can be sensitive to the update order of nodes and have difficulty in converging. Leung et al. proposed an improved label propagation method named LHLC by introducing scores to represent the transmission intensity of labels with the iterative process. However, the result is susceptible to the parameter of attenuation [16]. In addition, in order to improve the accuracy of community detection, some label propagation methods adopt the process of modularity optimization to get more robust results, but the running time and space complexity significantly increases [14, 15].

To improve the accuracy and robustness of label propagation, we propose a method by using the *α*-degree neighborhood impact for community detection, called NILP. Given a certain value of *α*, we firstly calculate the *α*-degree neighborhood impact of each node. Then, we arrange the nodes for updating process in ascending order on their *α*-degree neighborhood impact values. Thirdly, we update the label of each node asynchronously, and the new label is the one that has the maximum of the sum of weighted *α*-degree neighborhood impact. The main contributions of our method are as follows: (1) we propose a method to calculate the *α*-degree neighborhood impact, which can quantify the centricity of a node within its local link structure. (2) Our method takes the impact of neighborhood into consideration in the label update process, which makes it more robust than other label propagation algorithms. (3) Our method separates the process of calculating *α*-degree neighborhood impact and updating labels of nodes, which improves the running time efficiency. (4) A certain order of label updating is given which can expedite the convergence process.

The rest of the paper is organized as follows.  Section 2 introduces *α*-degree neighborhood networks, as well as the *α*-degree neighborhood impact formula.  Section 3 describes the working principle and steps of the proposed algorithm *α*-NILP in detail.  Section 4 presents the experimental results and the analysis. Finally, Section 5 concludes the paper.

## 2. *α*-Degree Neighborhood Impact

Given a network *G* = (*V*, *E*), where *V* is the set of nodes and *E* is the set of edges, and the task of network community detection is to find densely connected subgraphs in *G*. The label propagation method is applied here to implement automatic community detection [13]. Taking nodes as the basic computing units, we initialize every node with a unique label and let the labels propagate in a certain order through the network. In order to make densely connected nodes have the same labels, we take the local link structure into consideration. In this section, some related definitions are given as follows.

**Definition 1 (*α*-degree neighbor).**

Let *G* = (*V*, *E*) be an undirected network, where *V* is a set of nodes and *E* is a set of edges. Let *u*, *v* ∈ *V*. If the length of the shortest path from node *u* to *v* is *α*, then node *v* is called the *α*-degree neighbor of node *u*, denoted by $u\overset{\alpha }{\to }v$. $\Gamma (u)=\{v|v\in V\wedge u\overset{\alpha }{\to }v\}$ is the set containing all the *α*-degree neighbors of *u*.

It is obvious that the definition of *α*-degree neighbor is symmetrical, which means if node *u* is the *α*-degree neighbor of node *v*, then so is node *v* to node *u*. Particularly, node *u* is the 0-degree neighbor of itself.

**Definition 2 (*α*-degree neighborhood network).**

Let *G* = (*V*, *E*) be an undirected network with node *u*, *v* ∈ *V* and $V^{\prime }=\{v|v\in V\wedge u\overset{\epsilon }{\to }v\wedge 0\leq \epsilon \leq \alpha \}$. The spanning subgraph *G*′ = (*V*′, *E*′), which is composed of *V*′ and *E*′ = {〈*u*, *v*〉∣*u*, *v* ∈ *V*′∧〈*u*, *v*〉 ∈ *E*}, is called the *α*-degree neighborhood network of node *u*.

**Figure 1:**
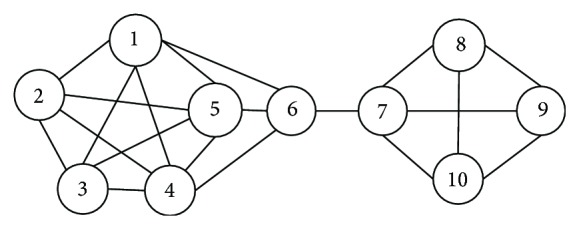
A sample network.

As shown in Figure 1, nodes 2–6, which are the neighbors of node 1 and are called its 1-degree neighborhood nodes, form the 1-degree neighborhood network of node 1 with all the incident edges of those nodes. Node 7 is a 2-degree neighbor of node 1, and the spanning subgraph composed of nodes 1–7 is a 2-degree neighborhood network of node 1. In general, we can view an *α*-degree network as a complete closed system constituted by an initiating center node and its surrounding counterparts and their incident edges. In this system, starting from a certain node *u*, we measure and analyze its local connection density via its *α*-degree neighbors and neighborhood network to yield the average degree of impact on all its surrounding nodes.

In a real network, a node affects its neighbors through its edges. In an unweighted network, a center node *u* wields precisely identical influence on its every neighbor. When the network is a weighted one, the degree to which surrounding neighbors will be affected by their center node is in proportion to the weight assigned to the incident edges. While a center node *u* influences all its neighbors, the center itself also absorbs impacts exerted by its neighbors. Due to the link path characteristics inherent in networks, the influence of a node on its 2-degree neighbors is the mean value of impacts on all its 1-degree neighbors. In the following, we give the calculation formula of the *α*-degree neighborhood impact.

**Definition 3 (*α*-degree neighborhood impact).**

Let *G* = (*V*, *E*, *λ*) be an undirected and weighted network *G* = (*V*, *E*, *λ*), where *V* is a set of nodes, *E* is a set of edges, and *λ* is the weight function of edges. The weight between nodes *i* and node *j* is *λ*_*ij*_  (*λ*_*ij*_ > 0), and 1 is the default value for the weight in an unweighted network. The formula for 0-degree neighborhood impact of a node is

(1) $$\begin{array}{c} VI_{x}^{\left(0\right)}=1, \end{array}$$

where *λ*_*ix*_ represents the weight of the edge between node *i* and node *x*. For node *x* to its *α*-degree neighborhood nodes (*α* ≥ 1), the impact formula is

(2) $$\begin{array}{c} VI_{x}^{\left(\alpha \right)}=\frac{\sum_{i\in \Gamma_{1}(x)}^{}\left(\lambda_{ix}\cdot VI_{i}^{\left(\alpha -1\right)}\right)}{\sum_{i\in \Gamma_{1}(x)}^{}\lambda_{ix}},\;\alpha >1. \end{array}$$

Given a network *G* = (*V*, *E*) and the parameter *α* ≥ 1, through recursive calculation, we can get the *α*-degree neighborhood impact scalar *VI* ^(*α*)^ = (*VI*_1_^(*α*)^, *VI*_2_^(*α*)^,…, *VI*_*n*_^(*α*)^) of each node.

**Figure 2:**
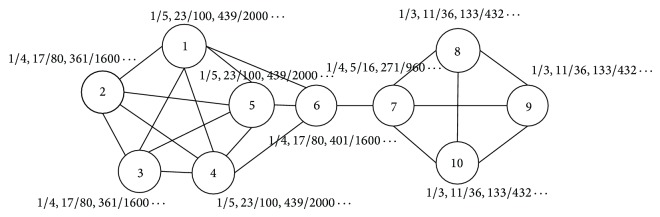
Average node impact in the sample network (*α* = 1, 2, 3).

The weights of the edges of the sample undirected network given in Figure 1 are considered as 1. As shown in Figure 2, the *α*-degree neighborhood impact of each node is calculated by formulas (1) and (2) in the sample network shown in Figure 1 with parameter *α* = 1, 2, and 3. For example, for node 7, the 1-degree neighborhood impact is 1/4, the 2-degree impact is 5/16, and the 3-degree impact is 271/960. We take *VI*_7_^(3)^ below as an example, illustrating the calculation procedure of 3-degree neighborhood impact. Consider

(3) VI7(3)=VI6(2)+VI8(2)+VI9(2)+VI10(2)4=VI11+VI41+VI51+VI714+VI71+VI91+VI1013  +VI71+VI81+VI1013+VI71+VI81+VI913 ×14=14VI11+14VI41+14VI51+54VI71+23VI81 +23VI91+23VI10(1)=271960.

We can find that as the value of *α* increases, the scanning range of the neighbors of a node gradually expands. The calculation of *α*-degree neighborhood impact fully considers every path whose end point is itself and the length is *α*. The effects of *α*-degree neighborhood of node *u* (including 1-degree neighborhood, 2-degree neighborhood,…, *α*-degree neighborhood) will spread along all possible paths and ultimately have a tangible influence on node *u*. Eventually, *α*-degree neighborhood impact of node *u* is the weighted average of all the (*α* − 1)-degree neighborhood impact of the neighbors of node *u*. For any node *u* in a network, the fact that its average *α*-degree neighborhood impact is comparably small indicates that nodes and edges in *α*-degree neighborhood network of node *u* are relatively dense, and the node *u* has strong centricity. Therefore, node *u* is less affected by its neighborhood, and the label of node *u* is more stable. The larger the average *α*-degree neighborhood impact of node *u* is, the sparser the links between nodes and edges are and the weaker the centricity that node *u* has. Thus in such case, for node *u*, the effect of its neighborhood is lager and the label is susceptible to change. In our method, all the nodes in network *G* are in ascending order on their *α*-degree neighborhood impacts, and we choose this order as the updating order of labels, which makes the updating order of labels relatively constant. In addition, the smaller the impact is, the earlier the node updates. We strive to avert label updating oscillation to facilitate convergence.

**Definition 4 (ratio of stable node).**

In the label updating process, after one iteration, the percentage of nodes possessing exactly identical labels as before is called the ratio of stable node. We can calculate the stable node ratio *p* as

(4) $$\begin{array}{c} p=\frac{N_{c}}{\left|V\right|}, \end{array}$$

where *N*_*c*_ is the number of nodes whose labels have no change in this round of iteration.

The stable node ratio *p* can be employed to measure the degree of convergence of our algorithm in the duration of label propagation.

## 3. Proposed Algorithm

Just like the original label propagation algorithm LPA, our algorithm based on *α*-degree neighborhood impact also iteratively updates labels according to a node traversal order and will eventually group nodes with the same label into the same community. The difference is that we introduce the impact values for each node and use it to determine the rankings of nodes and to update the node labels.

### 3.1. Label Updates

The method of updating label in algorithm *α*-NILP is based on the average impact of neighborhood nodes. When the label of node *u* needs to be updated, we use the following formula to determine its new label:

(5) $$\begin{array}{c} l_{u}^{\text{new}}=\underset{l}{\max}\underset{i\in N(u)}{\sum }\left(VI_{i}^{\left(\alpha \right)}\cdot \delta \left(l_{i},l\right)\right), \end{array}$$

where *N*(*u*) is a set of 1-degree neighbors of node *u* and *δ*(*i*, *j*) is the Kronecker function. If *i* = *j*, then *δ*(*i*, *j*) = 1; otherwise *δ*(*i*, *j*) = 0.

Therefore, the label of the 1-degree neighbor that exerts the greatest influence becomes the new label of node *u*. If there exist multiple choices of greatest neighborhood influence labels of node *u*, we randomly select a label as the new label of node *u*.

### 3.2. Algorithm Description

Given *α* ≥ 1, we can describe our algorithm *α*-NILP in the following steps.

**Step 1 .**

For any node *u* in a complex network *G* = (*V*, *E*), calculate *VI*_*u*_^(*α*)^ the average *α*-degree neighborhood impact of node *u*.

**Step 2 .**

According to the *α*-degree neighborhood impact *VI*_*u*_^(*α*)^, arrange the nodes in the network in an ascending order on the impact values to determine the updating order of node labels.

**Step 3 .**

For any node *u* ∈ *V*, assign it a unique label, and set the stable ratio *p* = 0.

**Step 4 .**

According to the determined updating order above, use formula (5) for updating labels of all the nodes.

**Step 5 .**

Calculate stable ratio *p*_1_ of the current round of label update.

**Step 6 .**

Compare the value of *p* and *p*_1_; if *p*_1_ ≥ *p*, then *p* : = *p*_1_, and go to Step 4 to continue to update the node labels; otherwise, stop updating, and perform rollbacks of all the node labels to revert them to their previous states.

**Figure 3:**
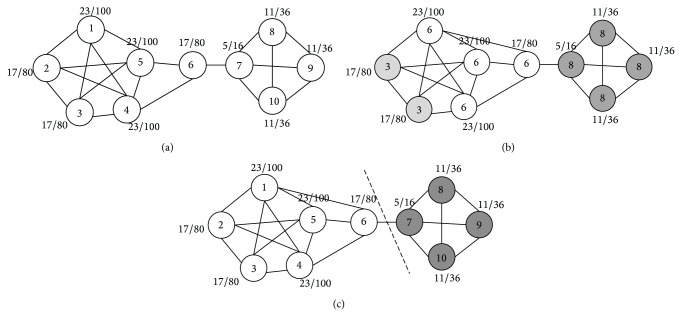
The process of label propagation by using algorithm NILP to detect community structure on the sample network.

Figure 3 illustrates the process of community detection using algorithm NILP in the above example network when *α* = 2. In Figure 3(a), in the sample network, each node is marked with a unique label, and the 2-degree neighborhood impact values are labeled beside the nodes. According to the ascending sort order of the impact values, the nodes update order is determined as 5 → 1 → 4 → 2 → 3 → 6 → 7 → 8 → 9 → 10. Node 5 is the first one for label update, using formula (5) to decide the new label, and the result for adjacent neighborhood node 6 has the greatest influence on it, so we change the label of node 5 to the node number of its neighbor, in case 6. Next, we update all the nodes sequentially.  Figure 3(b) is the result of the divided community which is updated at the end of the first round of label propagation. After the first round of label update process completed, with the stable ratio of the current node being *p*_1_ = 0.3, we are supposed to update labels in accordance with the above order in the next round of node label update process. The algorithm continues to run until the stable ratio no longer rises.  Figure 3(c) shows the final results of our algorithm on detecting communities on the sample network.

Algorithm NILP is different from other label propagation based algorithms. First, NILP limits the scope of impact that nodes can exert on their neighbors to a variable *α*, and it differs from the attenuation degree setting in the label propagation process of LHLC, rendering it feasible for nonattenuation propagation in local areas in real life. Such as a network of friends, only a limited number of people within the scope of the friends will be in the same circle of friends. When the information of insiders' interest has been released, the information exchanges along the route of various relationships to attain the goal of information sharing, while outsiders are mostly not likely to disseminate such information because they are not interested in it. Secondly, NILP calculates the mean value of impact for each node in the scanning range of *α*-degree neighborhood and fully takes its *α*-degree neighborhood network structure into account, which improves the efficiency of the process of label propagation. Third, the mutual influence between nodes is an objective existence, independent of the label propagation, so the node neighborhood impact and the label iterative update process are separated. Due to the fact that label propagation proceeds with nodes affecting each other, the process of node update must be based on the value of average neighborhood impact. Finally, according to the size of neighborhood impact, NILP updates all the nodes in ascending order and makes the process of updating labels more definite instead of more randomized. In an unweighted network, when *α* → *∞*, all the neighborhood impact of nodes tends to be the same. So any node update order can be applicable to the label propagation process. Therefore, for the unweighted network, formula (5) can be simplified as

(6) $$\begin{array}{c} l_{u}^{\text{new}}=\underset{l}{\max}\underset{i\in N\left(u\right)}{\sum }\delta \left(l_{i},l\right). \end{array}$$

At this point, NILP algorithm becomes the original label propagation algorithm LPA. Hence, we can draw the conclusion that LPA is merely a simple case of our *α*-degree neighbors label propagation algorithm NILP.

### 3.3. Complexity Analysis

In this subsection, we analyze and compare both time and space complexity of various label propagation based algorithms *α*-NILP, LPA, LPAm, and LHLC. The pertinent data is shown in Table 1. In terms of time complexity, our algorithm *α*-NILP consists of three parts which are the calculation of *α*-degree neighborhood impact, the node sorting process, and the label propagation process. In the calculation of impact values, our algorithm needs to traverse all the nodes in the network and the 1-degree neighbors of all the nodes, so the time complexity is *O*(*αm* + *n*), where *m* and *n* are, respectively, the number of edges and nodes in the network. In the sorting process, we adopt quick sort algorithm and the time complexity is *O*(*n*log⁡*n*). The time complexity of the label propagation process is *O*(*n*log⁡*n*). Therefore, the overall time complexity is *O*(*n*log⁡*n*) when *O*(*m*) = *O*(*n*) in a sparse scale-free network.

Then, we analyze the space complexity of our *α*-NILP algorithm. Because the algorithm creates *n* nodes and *n* initial communities, we use adjacency lists to describe the 1-degree relationship between nodes and the correspondence between nodes and communities, which occupies *O*(2*m* + *n*) and *O*(*n* + *n*) space, respectively, and amounts to the total space complexity of *O*(*n*).

In summary, in the case of the same time complexity, LPA, LHLC, and *α*-NILP have lower space complexity. This is because these algorithms run without using adjacency matrix, which leads to the decline of the volume of data involved in the creating, reading, and manipulating process. The running time elapsed also dwindles due to the reduction in the space complexity, implying that the above three algorithms also run faster.

## 4. Experimental Results and Analysis

In this section, we evaluate the performance of the proposed algorithm *α*-NILP through experiments. Our algorithm is implemented using ANSI C++. All the experiments were conducted on a PC with 3.20 GHz processors and 4.0 GB memory.

### 4.1. Data Sets

To evaluate the performance of our algorithm, we use the following three real-world networks.

*Zachary's Karate Club Network.* A network of social relations between members of an American university karate club (http://networkdata.ics.uci.edu/data.php?id=105) is composed of 105 vertexes and 882 edges, where each vertex represents a club member and an edge denotes the fact that the two members, assumed as friends rather than mere acquaintances by us, contact each other frequently. Due to internal disputes, the club splits into two groups, which is its real network community structure.

*NCAA College-Football Network*. The network of American football games between Division IA colleges during Regular Season Fall 2000 (http://networkdata.ics.uci.edu/data.php?id=5) is composed of 115 vertexes and 1,232 edges, in which each vertex corresponds to an American college football team and each edge represents two corresponding teams played a game during Regular Season Fall 2000. All the teams are divided into eleven conferences and five independent teams.

*Books about US Politics*. The network of books about recent US Politics sold by the online bookseller is composed of 105 vertexes and 882 edges, in which each vertex corresponds to an US Politics book and each edge represents the frequent copurchasing of two corresponding books.

*DBLP Coauthorship Network*. A weighted network of authorship in four research fields (i.e., DB, IR, DM, and ML) extracted from the DBLP computer science bibliographical dataset is composed of 28,702 vertexes and 66,832 edges, in which each vertex corresponds to a distinct author who has published more than twenty papers and each edge represents their coauthor relationship. The weight of an edge denotes the number of papers coauthored by these two authors.

Meanwhile, we utilize the tool developed by Lancichinetti et al. [17] to generate several synthetic networks and divide them into two groups based upon the number of nodes in networks, with the nodes number of one group being 1000 and the other group 10000. Each group comprises 15 networks, with their mixing coefficient ranging from 0.1 to 0.8 at a step size of 0.05. To further evaluate the performance of our method, we also run our algorithm on networks of different number of nodes, including 1000, 5000, 25000, 5000, 100000, 250000, and 500000, with the mixing coefficient being 0.3.

### 4.2. Analysis of the Influence of Parameter *α*

**Figure 4:**
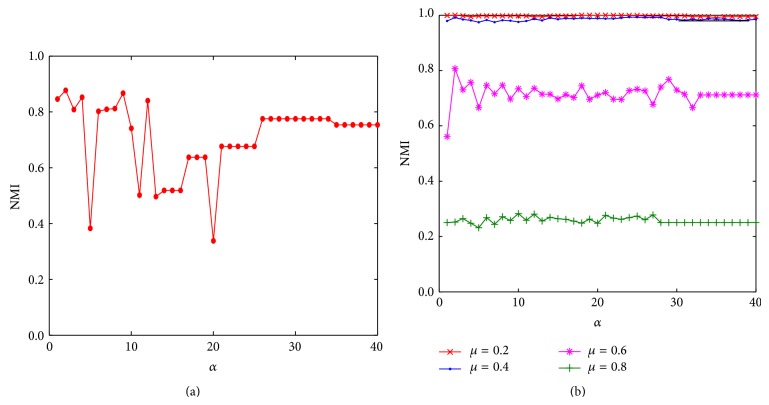
The achieved NMI values of our algorithm varying with the parameter *α* in a real network Football and the synthetic networks with *n* = 1000.

To compare the impacts of different values of *α* on the performance of our algorithm, we conduct our experiment on the benchmark Football dataset and fifteen 1000-node synthetic LFR networks with their mixing coefficients varying from 0.1 to 0.8 at an increment interval of 0.05. Setting the values of *α* from 1 to 40, when detecting communities in the real network Football and the synthetic networks, the NMI values of our algorithm are shown in Figures 4(a) and 4(b).

As shown in Figure 4(a), in the real Football network, when *α* = 2, the highest NMI value is obtained, indicating that the results are the closest to the correct ones. When *α* = 5, 11, 13, 20, NMI value fluctuates drastically, which is because, under these values, the obtained label update order and the neighborhood impacts of nodes divide the real network into several large-scale communities, and thus NMI is significantly reduced. This indicates that the link structure of a real network has some randomness; thus a label propagation based algorithm running in these networks for community detection is more sensitive to the traversal order of nodes.  Figure 4(b) shows the experimental results on the 1000-node synthetic networks, and we can find that, compared with the real network, this algorithm is more stable on the synthetic networks. When the mixing coefficient *μ* = 0.2, 0.4, or 0.6, *α* = 2 can always yield the maximum NMI value. For the network of mixing coefficient being 0.8, the value of NMI is not a maximum when *α* = 2, but it is very close to the maximum value. A large number of experiments show that, in most cases, the community-dividing results of the proposed algorithm NILP are optimal or near-optimal when *α* = 2. Therefore, all the subsequent parts of our experiment were conducted using 2-NILP for experimental analysis.

### 4.3. Evaluation on Real Networks

**Figure 5:**
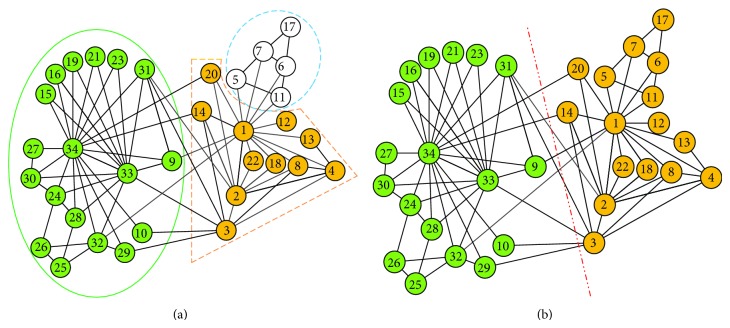
The comparison of results detected by algorithms LPAm and 2-NILP in Zachary's Karate networks.

First, we analyze the results of the algorithms NILP and LPAm in Zachary's Karate network, as shown in Figure 5. In Figure 5(a), the detection result of algorithm LPAm is given, in which the network is divided into three communities, while algorithm 2-NILP divides the network into two communities, which is exactly the real situation, just as the ground truth shown in Figure 5(b). Comparing the two figures, we can tell that the most notable difference lies in whether the node set {5,6, 7,11,17} is seen as a separate community or not. As can be seen from the graph, the structure of the subgraph composed of the nodes {5,6, 7,11,17} is relatively stable, and {5,6, 7,11} are closely connected with node 1, so the node set {5,6, 7,11,17} should belong to the community which node 1 belongs to. Algorithm LPAm adopts local modularity optimization principle but does not find the optimal division of communities, while our 2-NILP algorithm discovers the network structure by calculating the local neighborhood impacts and analyzing density of local areas. Although the optimal partition does not necessarily have the largest network module values, it is more effective in detecting the intrinsic community structure of networks. The NMI values that we obtained from the experiments of the four different kinds of label propagation algorithms, namely, LPA, LPAm, LHLC, and 2-NILP, on network Zachary's Karate and Football are listed in Table 2. As can be seen from Table 2, our algorithm 2-NILP achieved the best results in terms of accuracy, and this is also almost true for LPAm which has decent accuracy. However, earlier proposed label propagation algorithms LPA and LHLC have lower accuracy due to their update processes not being well controlled.

In the following, we will analyze the result of our NILP algorithm on a real DBLP coauthorship network. Since the network DBLP does not provide a standard result which can be used to compare, we assess the correctness of the obtained communities by referring to the data source of the network. The proposed method detected 3,466 communities of different sizes in this network.  Table 3 lists the five real communities detected. Due to the limitation of space of our paper, only seven members are listed for each community. As can be seen from Table 3, the Community [1] and Community [2] are experts and scholars in the field of data mining in which Philip S. Yu and Jiawei Han are regarded as their leading figures, respectively. Community [8] is composed of the experts and scholars in database who are from InfoLab laboratory at Stanford University. Community [188] comprises experts and scholars from CMU in the field of machine learning and Community [346] is constituted by experts and scholars in the field of information retrieval. It can be observed that scientists from one community, detected by our algorithm, are often in the same realm of research, which accounts for their frequent academic collaboration. In the same field, usually there are multiple communities which are formed from different work teams. In a team, often there is a common or similar research direction and long-term cooperation, while different work teams will rarely have chance to collaborate. Consequently, the community detection result obtained from DBLP via the proposed algorithm is sound and accurate.

### 4.4. Evaluation on Synthetic Networks

**Figure 6:**
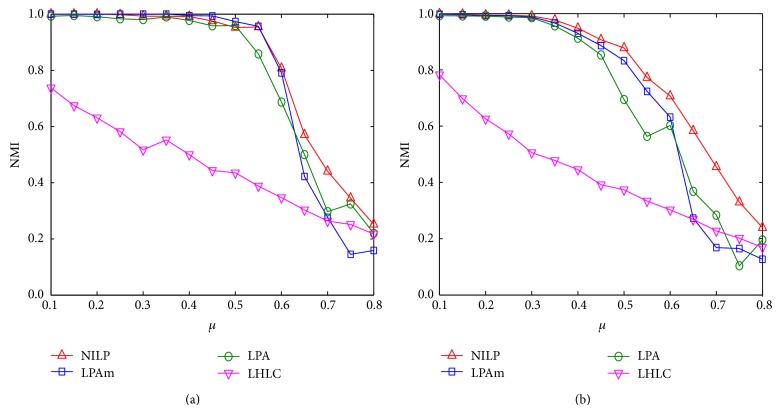
The NMI values varying with the mixing coefficient achieved by four label propagation algorithms on the synthetic networks.

We also evaluate the performance of our algorithm on synthetic networks.  Figure 6 illustrates the comparison of accuracy for community detection of four label propagation based algorithms LPA, LPAm, LHLC, and 2-NILP. The mixing coefficients of the 1000-node synthetic networks in Figure 6(a) and 10000-node networks in Figure 6(b) both range from 0.1 to 0.8. It can be observed that the accuracy of LHLC is relatively low compared with the other three algorithms. Algorithms LPA, LPAm, and NILP have higher values of NMI. When the number of nodes is 1000, as shown in Figure 6(a), the accuracy of 2-NILP is obviously better than that of the algorithm LPA. When mixing coefficient is less than 0.55, 2-NILP has equal accuracy with the algorithm LPAm, while when mixing coefficient is greater than 0.55, 2-NILP is significantly better than LPAm. When the number of nodes is 10000, as shown in Figure 6(b), the accuracy of our algorithm 2-NILP is superior to the other three algorithms.

### 4.5. Running Time Comparison

**Figure 7:**
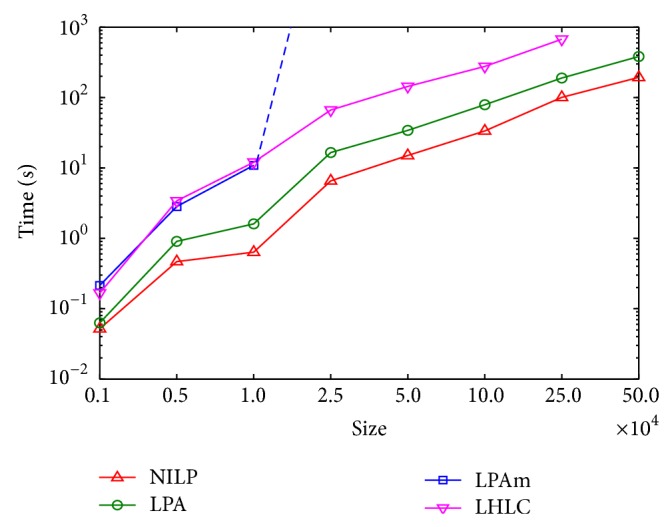
Running time comparison of four label propagation based algorithms.

In order to compare the efficiency of the four algorithms above, we continue to conduct experiments on the synthetic networks, and the experimental results are shown in Figure 7. In this experiment, we selected the network whose mixing coefficient is 0.3 and the number of nodes is 1000, 5000, 10000, 25000, 50000, 100000, 250000, and 500000. As can be seen from Figure 7, in the same circumstances, running time of our algorithm NILP should be less than that of other three algorithms. This is because NILP calculates the *α*-degree neighborhood impact of each node and updates the labels according to the degree of impact, and the final label is closely related to its impact; thus NILP algorithm can make the node labels achieve their stability more easily. As a result, algorithm NILP needs less time compared with the other three algorithms. Owning to the tremendous space cost incurred at runtime, when the number of nodes exceeds 10000, algorithm LPAm fails to proceed to its completion in reasonable time.

## 5. Conclusion

In this paper, a novel label propagation based algorithm, called NILP, is proposed for community detection in networks. Based on the link structure in networks, our method introduces measurement of node *α*-degree neighborhood impact, which fully considers the impact that nodes have on their neighbors in order to determine the updating order of node labels. The proposed method improves the accuracy and efficiency of community detection and reduces the memory consumption. The result of our method is prominent in various kind of networks. It is suitable for community detection and evolution analysis of dynamic networks, especially with a large number of online social networks.

## Figures and Tables

**Table 1:** The comparison of time and space complexity of four algorithms LPA, LPAm, LHLC, and *α*-NILP based on label propagation (*n* is the number of nodes in the network).

Label propagation based algorithm	LPA	LPAm	LHLC	*α*-NILP

Time complexity	*O*(*n*log⁡*n*)	*O*(*n*log⁡*n*)	*O*(*n*log⁡*n*)	*O*(*n*log⁡*n*)

Space complexity	*O*(*n*)	*O*(*n* ^2^)	*O*(*n*)	*O*(*n*)

**Table 2:** The accuracy comparison of various label propagation algorithms in networks with ground truth of community structure.

Real networks	LPA	LPAm	LHLC	2-NILP

Zachary's Karate	9.6*E* − 05	0.825518	0.422542	**1**

NCAA College-Football	0.485261	0.828798	0.404785	**0.877295**

Books about US Politics	0.0310231	0.17699	0.31861	**0.452619**

**Table 3:** The accuracy comparison of various label propagation algorithms in networks with ground truth of community structure.

Community [1]	Community [2]	Community [8]	Community [188]	Community [346]
Philip S. Yu	Jiawei Han	Hector Garcia-Molina	Tom M. Mitchell	Douglas W. Oard
Haixun Wang	Xifeng Yan	Jennifer Widom	Reid G. Smith	Anton Leuski
Charu C. Aggarwal	Dong Xin	Jeffrey D. Ullman	Louis I. Steinberg	G. Craig Murray
Wei Fan	Deng Cai	Yannis Papakonstantinou	Mark A. Jones	J. Scott Olsson
Kun-Lung Wu	Hong Cheng	Rajeev Motwani	Van E. Kelly	Jianqiang Wang
Zhongfei (Mark) Zhang	Xiaofei He	Inderpal Singh Mumick	Sen Slattery	David S. Doermann
Bugra Gedik	Xiaolei Li	Vagelis Hristidis	Gilles M. E. Lafue	Kareem Darwish
*⋯*	*⋯*	*⋯*	*⋯*	*⋯*
